# Community Preventive Services Task Force Issues 2013 Annual Report to Congress

**Published:** 2013-07-12

The Community Preventive Services Task Force recently posted its *2013 Annual Report to Congress and to Agencies Related to the Work of the Task Force* on its website. The report, which focuses on cardiovascular disease, is available at http://www.thecommunityguide.org/annualreport/index.html.

Established in 1996 by the U.S. Department of Health and Human Services, the task force is an independent, nonfederal, unpaid panel of public health and prevention experts whose members are appointed by the Director of CDC. The task force provides information for a wide range of decision makers on programs, services, and policies aimed at improving population health. Although CDC provides administrative, research, and technical support for the task force, the recommendations developed are those of the task force and do not undergo review or approval by CDC.

